# Biologically relevant effects of mRNA amplification on gene expression profiles

**DOI:** 10.1186/1471-2105-7-200

**Published:** 2006-04-11

**Authors:** Rachel IM van Haaften, Blanche Schroen, Ben JA Janssen, Arie van Erk, Jacques JM Debets, Hubert JM Smeets, Jos FM Smits, Arthur van den Wijngaard, Yigal M Pinto, Chris TA Evelo

**Affiliations:** 1 BiGCaT Bioinformatics, University of Maastricht and Technical University Eindhoven, Maastricht, The Netherlands; 2 Experimental and Molecular Cardiology/ CARIM, University of Maastricht, Maastricht, The Netherlands; 3 Department of Pharmacology and Toxicology/ CARIM, University of Maastricht, Maastricht; 4 Department of Genetics and Cell Biology, University of Maastricht, Maastricht

## Abstract

**Background:**

Gene expression microarray technology permits the analysis of global gene expression profiles. The amount of sample needed limits the use of small excision biopsies and/or needle biopsies from human or animal tissues. Linear amplification techniques have been developed to increase the amount of sample derived cDNA. These amplified samples can be hybridised on microarrays. However, little information is available whether microarrays based on amplified and unamplified material yield comparable results.

In the present study we compared microarray data obtained from amplified mRNA derived from biopsies of rat cardiac left ventricle and non-amplified mRNA derived from the same organ. Biopsies were linearly amplified to acquire enough material for a microarray experiment. Both amplified and unamplified samples were hybridized to the Rat Expression Set 230 Array of Affymetrix.

**Results:**

Analysis of the microarray data showed that unamplified material of two different left ventricles had 99.6% identical gene expression. Gene expression patterns of two biopsies obtained from the same parental organ were 96.3% identical. Similarly, gene expression pattern of two biopsies from dissimilar organs were 92.8% identical to each other.

Twenty-one percent of reporters called present in parental left ventricular tissue disappeared after amplification in the biopsies. Those reporters were predominantly seen in the low intensity range.

Sequence analysis showed that reporters that disappeared after amplification had a GC-content of 53.7+/-4.0%, while reporters called present in biopsy- and whole LV-samples had an average GC content of 47.8+/-5.5% (P <0.001). Those reporters were also predicted to form significantly more (0.76+/-0.07 versus 0.38+/-0.1) and longer (9.4+/-0.3 versus 8.4+/-0.4) hairpins as compared to representative control reporters present before and after amplification.

**Conclusion:**

This study establishes that the gene expression profile obtained after amplification of mRNA of left ventricular biopsies is representative for the whole left ventricle of the rat heart. However, specific gene transcripts present in parental tissues were undetectable in the minute left ventricular biopsies. Transcripts that were lost due to the amplification process were not randomly distributed, but had higher GC-content and hairpins in the sequence and were mainly found in the lower intensity range which includes many transcription factors from specific signalling pathways.

## Background

Gene expression microarrays have become well established technology with which the expression of 10,000's of genes can be measured simultaneously on a single glass slide. The primary limitation of large-scale gene expression studies has always been the requirement for relatively large amounts of input RNA. Reduction of input RNA by use of an amplification step can greatly expand the possibilities of gene expression studies, towards small biopsies from small animals like e.g. rodents and even laser capture micro dissection material. RNA amplification strategies have been reported in the past few years, in which a T7-based linear in vitro transcription is most commonly used [1-3]. In general, these studies describe reproducible results with high correlations between amplified and non-amplified RNAs derived from a common pool of high-quality RNA. The importance of high quality of the starting total RNA for amplification is often stressed [4,5]. However, it is well-known that small tissue samples, especially when derived from organs that have low RNA contents, do not yield high-quality RNA [6]. Therefore, we examined the applicability of the RNA amplification technique in very small, possibly lower-quality biopsy-RNA of left ventricular (LV) rat heart tissue, by evaluating their gene expression patterns. The biopsies were taken according to a newly developed methodology that enables repeated sampling of cardiac biopsies in rats in-vivo. Gene array analysis was performed in order to investigate how the gene expression profiles of the biopsies relate to the profiles found in their parental LVs.

## Results

### Evaluation of left ventricular contractility after taking biopsies

**Figure 1:**
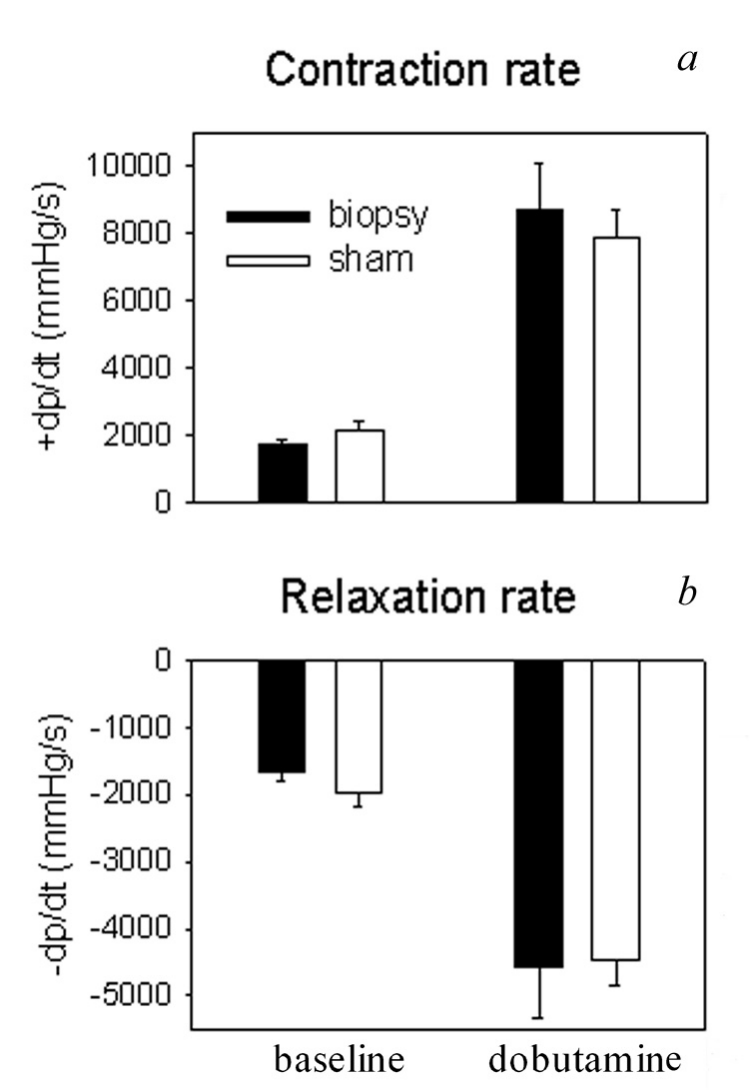
Two weeks after surgery, contraction rate (+dP/dt) (*a*) and relaxation rate (-dP/dt) (*b*) of 6 rat hearts from which biopsies were taken were compared to data obtained from 6 sham-operated rats. Data were obtained by direct LV pressure measurements using a micro-tip pressure transducer (Millar Instruments, Tx, USA) inserted into the left ventricle. Data were obtained at baseline as well as during dobutamine-stimulated conditions.

The taking of small biopsies of LV tissue might affect cardiac function. To examine this possibility we compared cardiac contractility in 6 biopsied and 6 sham-operated rats. The LV biopsy procedure was not associated with mortality; all rats survived the observation period of 14 days. In addition, the biopsy procedure did not affect cardiac contractility and relaxation, the two major determinants of cardiac function (Figure 1).

### RNA isolation, amplification and pitfalls

**Figure 2:**
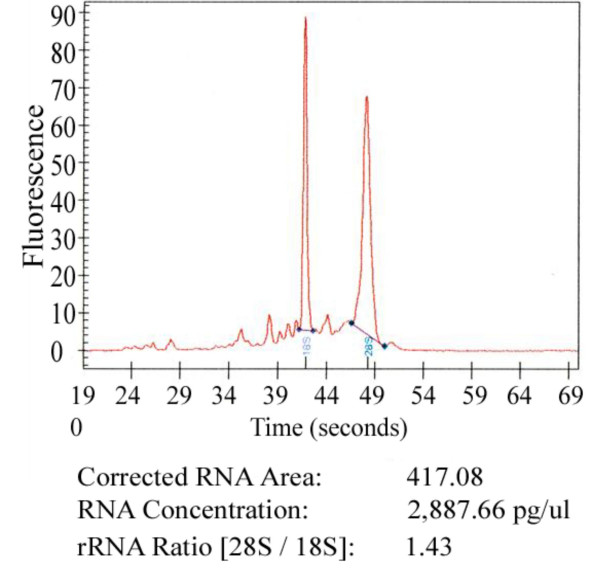
Representative Bioanalyser Picochip plot of RNA isolated from one biopsy, with a 28S/18S ratio of 1.43.

Two rats were used for this experiment. From each LV 3 biopsies were taken. We were able to successfully isolate RNA from 5 out of 6 biopsies (biopsy #2–3 from LV #1; biopsy #4–6 from LV #2), and from the two parental LV samples (LV #1 and #2). Figure 2 shows a representative example of a Bioanalyser Picochip-plot of RNA isolated from a rat heart biopsy. Each biopsy approximately yielded 100 ng of total RNA showing some degradation of 28S ribosomal RNA. Biopsy #1 failed to yield RNA because during surgery the needle did not contain cardiac tissue rather coagulated blood.

We used 30 ng total RNA of each biopsy sample as input for first round amplification, and the total amplified cRNA products were directly biotin labelled in a second standard Affymetrix amplification reaction. Amplification efficiency was then quantified by OD_260_. Four out of 5 labelled products contained more than 200 ng cRNA (ranging from 300 to 750 ng indicating a 1000–2500 fold amplification), which was sufficient to hybridize on an Affymetrix GeneChip microarray. Only biopsy #3-cRNA yield was not sufficient (156 ng). As a result 4 out of 6 biopsies (#2, #4, #5, and #6) plus two parental LVs were hybridized on Affymetrix GeneChip microarrays. Length of cRNA ranged from 100 to 5000 base pairs as determined by Bioanalyser Nanochip.

### TEST-3 chips and rat 230A GeneChips

A TEST-3 Affymetrix chip was run for each sample (2 parental LVs and 4 LV biopsies) to assess cRNA quality on a transcript level, after biotin labelling of parental LVs #1 and #2 starting from 5.8 μg total RNA, and after amplification and labelling starting from 30 ng of total RNA of LV biopsies #2, #4, #5 and #6. Next, the 4 LV biopsy- and the 2 parental LV-cRNAs were hybridized on rat 230 A GeneChips. Biopsy #5 was excluded from further analysis because of aberrant, low quality control measures on TEST3 and 230 A chips.

Consequentially, for the determination of gene expression profiles we obtained expression results from the two parental LVs and from three LV biopsies (one biopsy of LV#1 and two biopsies of LV#2)

### Real-time PCR analysis indicates amplification efficiency

**Figure 3:**
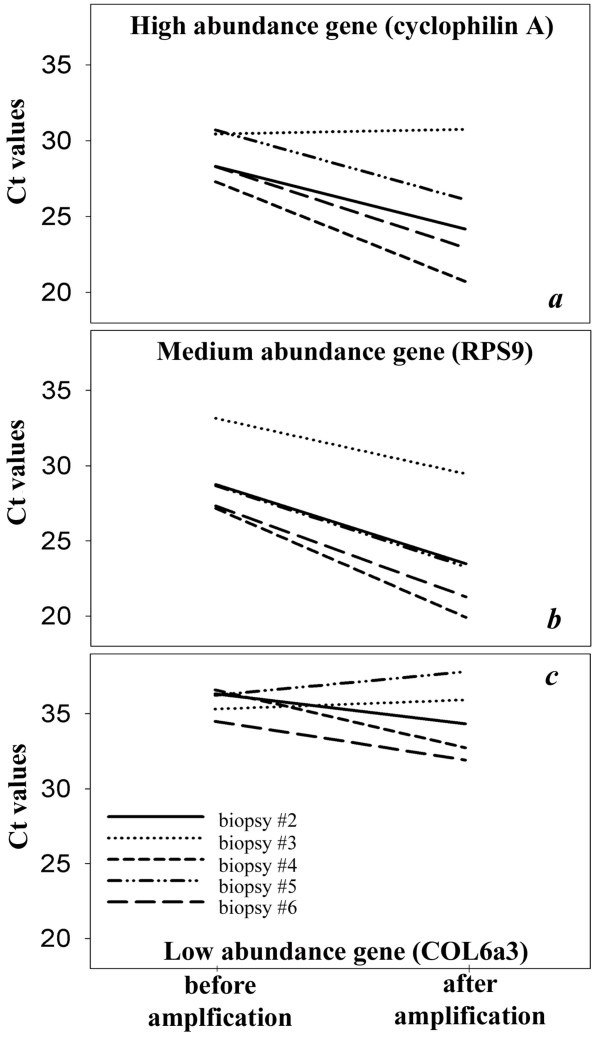
Quantitative real-time PCR (qPCR) of biopsy RNA before and after amplification using a high (cyclophilin A), medium (ribosomal protein S9, RPS9) and low (collagen VI alpha 3, COL6a3) abundance transcript. Material for the 'before amplification' qPCR was obtained from cDNA synthesis from total biopsy RNA; material for the 'after amplification' qPCR was obtained from cDNA synthesis from first round cRNA. After amplification, biopsy #3 yielded aberrant Ct values for all 3 genes; biopsy #5 only for COL6a3.

Quantitative real-time PCR analysis using a high-, a medium, and a low-abundance gene transcript discriminated between well- and badly amplified cRNA samples (Figure 3, panel *a *to *c*). In 3 out of 5 samples (biopsy #2, #4 and #6) it was observed that the number of PCR cycles needed to detect these 3 gene transcripts decreased after amplification. This indicates that these gene products were amplified successfully and confirmed the good quality of cRNAs, as seen after test chip and full chip analysis. The relative gene expression ratios between the biopsies were preserved after amplification. However in biopsy #3, the three gene transcripts yielded aberrant Ct values after amplification; their high Ct values indicated that these genes had not been amplified properly. For the same reason, amplification data from LV biopsy #5 were aberrant for the low abundance gene COL6a3.

These data are consistent with the results of the gene microarray experiment, in which only LV biopsies #2, #4 and #6 yielded gene expression data.

### Quality of results

In order to assess the quality of our biopsy samples on microarray, we evaluated the Average Relative Standard Deviation (ARSD) of the signal values, given by



where:

n_PS _= number of probe sets

sd_i _= standard deviation of the signal values in an experimental group for probe set i

 = average signal value in an experimental group for probe set i

Next to the biopsy group (n = 3) and the heart sample group (n = 2) of this study, we also looked at another biopsy study not yet published, consisting of three groups of rats (n = 4; n = 6 and n = 4) that were evaluated on the Affymetrix GeneChip^® ^Rat Genome 230 2.0 array. In addition we compared our results with a study by Schweitzer et al. downloaded from the Gene Expression Omnibus (GEO) [7]; GEO accession no. GSE2690[8]. Seventy-two male Sprague-Dawley rats were separated into three groups having different access to physical exercise. RNA was used from 12 heart tissue samples per group, the samples were pooled and applied to four Affymetrix GeneChip^® ^Rat Genome 230 2.0 arrays per group.

We selected probe sets (n = 4256) with a present call on all 31 arrays. To compensate for the different scanner and algorithm parameters used, we first took the log values of the signals, and subsequently performed a quantile normalization [9]. Since the experiments of this study were done on a 230 A array, we ignored the 230 B part of the 230 2.0 arrays.

As shown in Table 1, the ARSD values of both biopsy studies are within two standard deviations of the mean ARSD. Even when the pooled arrays of the Schweitzer study, which obviously have a smaller ARSD, are included the ARSD values are within two standard deviations of the mean ARSD. In addition, if we regard the arrays as vectors in a 4256 dimensional space, we can calculate distances between them, e.g. the Euclidean distance as we did in this case. The average distance between the probesets present on all the arrays within each experimental group are given in the rightmost column of Table 1. The average Euclidean distance in the current study is of the same order as in the two additional studies: for the biopsy group it is somewhat larger and for the parental hearts it is slightly smaller.

### Amplification results in non-random loss of gene detection

Gene array analysis was performed in order to investigate how the gene expression profiles of the LV biopsies relate to the profiles found in their parental LVs. The analysis firstly showed that parental LV #1 and #2 had 99.6 % identical gene expression (with fold change less then 2), indicating, as expected, that there was very little difference between those two rats. Gene expression patterns of LV biopsy #4 and #6 that were originally obtained from the same parental LV (#2), were for 96.3 % identical. Gene expression pattern of LV biopsy #2 was 92.8 % identical to the gene expression patterns of LV biopsy #4 and 6, indicating that biopsies obtained from dissimilar LVs yield comparable gene expression patterns.

**Figure 4:**
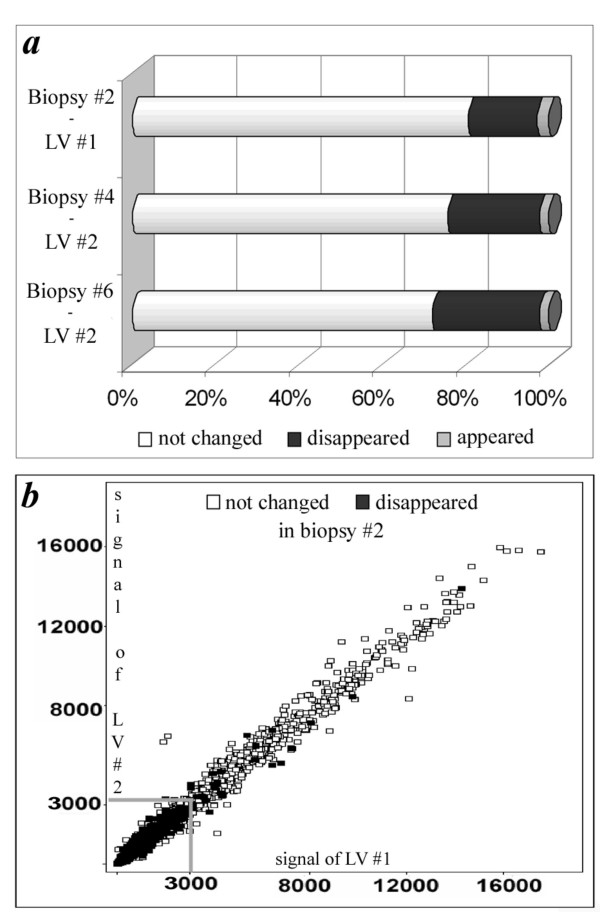
Gene array data analysis. (a) Summary of gene array characteristics, as described in the results section "Amplification results in non-random loss of gene detection". Low intensity means a signal of less than 3000 on the Affymetrix gene chip. (b) Signal intensity plot in which every gene is represented by a square. Signals of parental LVs #1 and #2 are similar, and gene expression data of LV biopsy #2 are visualized in the squares as 'not changed' or 'false negatives'. Biopsy reporters predominantly disappear in the lower expression region of their parental LVs, as exemplified by LV biopsy #2. Data on LV biopsy #4 and 6 are similar.

To further explore the information yielded by the two types of samples, we compared gene array characteristics of parental LVs and LV biopsies (Figure 4a). On average, 76 % of reporters detected in the parental LV samples #1 and #2 were also detected in LV biopsies #2, #4 and #6 (based on present call in Affymetrix Mas5.0 software). We compared the differential expression of parental LV#1 versus LV#2 with the differential expression of LV biopsy #2 versus LV biopsy #4 and 6 respectively, with a threshold level of 2-fold change. Of the reporters with present calls in both samples, 96 % did not differ between LV biopsy #2 and LV biopsies #4 and #6 respectively, whereas 4 % of reporters that were differentially expressed between the biopsies were not differential between their parental LVs.

Approximately 21 % of the reporters called present in parental LVs disappeared after amplification of mRNA of the LV biopsies (false negatives) while 3 % of reporters called absent in the parental LVs appeared after amplification in the LV biopsies (false positives) (Figure 4a). False negatives were predominantly (99.5% of total) observed in the low intensity range, which means reporters with signal less than 3000 in both parental LVs (Figure 4b).

**Figure 5:**
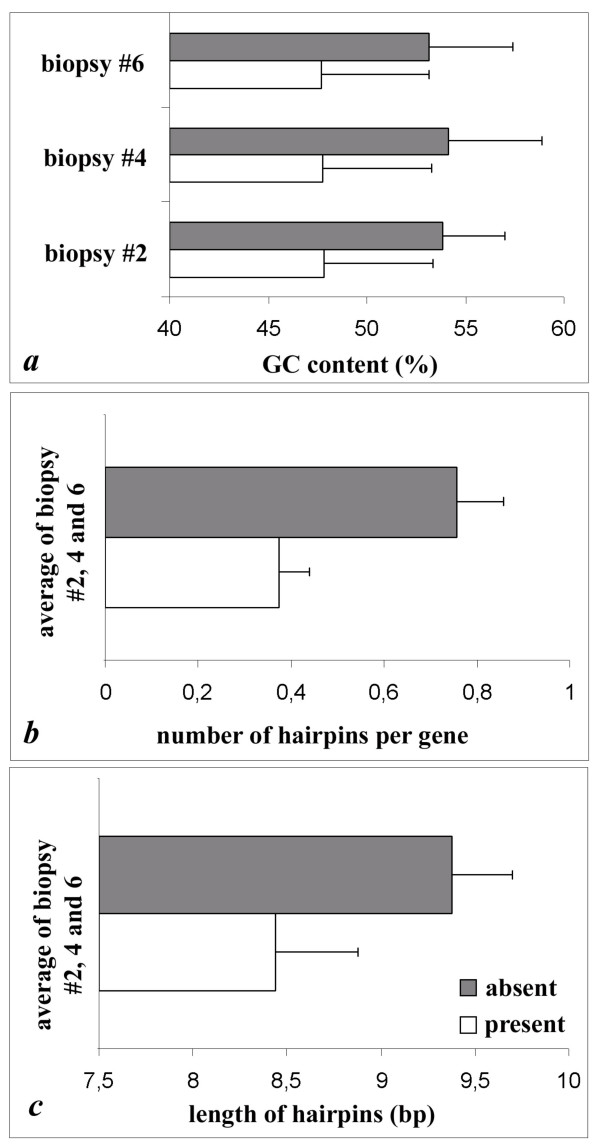
Reporters that were not detected after amplification ■ had a significantly higher mRNA GC-content (53.7% ± 4.0 *vs*. 47.8% ± 5.5, *P <*0.001) (a), and contained significantly more (***b***) and longer (***c***) hairpins as compared to representative control reporters present before and after amplification *o *(0.76 ± 0.07 *vs*. 0.38 ± 0.10 hairpins per gene resp., *P *< 0.01; and 9.4 base pairs (bp) ± 0.3 per hairpin *vs*. 8.4 bp ± 0.4 resp., *P *< 0.05. A hairpin must contain 7 or more base pairs. The compared reporters were taken from the same region of expression (same signal intensities).

This detection failure turned out to be significantly dependent on GC content and hairpin formation (Figure 5). Reporters that disappeared after amplification had a GC-content of 53.7% ± 4.0, while reporters called present in LV biopsy- and parental LV-samples had an average GC content of 47.8% ± 5.5 (P <0.001) (Figure 5a). This was also true for the reporters that were in the higher expression ranges (>3000), where false negatives had a GC content of 53.7% ± 4.2 compared to the 48.3% ± 5.4 for all reporters. In the lower intensity range (<3000) the difference in GC content between reporters that disappeared after amplification and reporters with a present call in both the parental LVs and LV biopsies is even larger, being 50.5% ± 6.1 and 43.4% ± 6.9 respectively.

**Figure 6:**
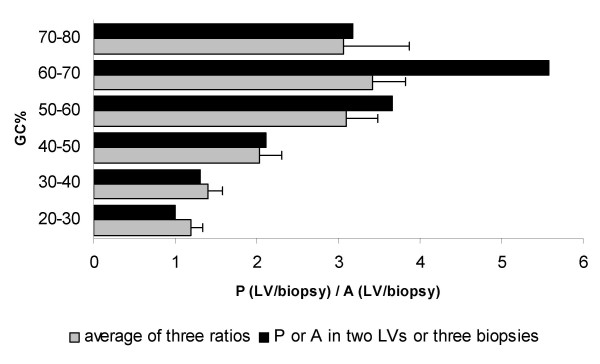
Ratio between present calls (heart/biopsy) and absent calls (heart/biopsy) calculated for the different GC%. GC% was subdivided in groups of 10%. Grey bars indicate the average ( ± SEM) of the three comparisons between parental LVs and LV biopsies; black bars represent the ratio of detection calls when the two parental LVs or all three LV biopsies have the same detection call.

Comparison of the detection calls between parental LVs and LV biopsies showed that the ratio between present calls (heart/biopsy) and absent calls (heart/biopsy) is higher than 1 for all GC contents (Figure 6). Figure 6 also shows that this ratio increases when the GC% of the reporters increases (with a maximum at GC content between 60 and 70 %),

The false negatives in the higher expression range were predicted to form significantly more (0.76 ± 0.07 versus 0.38 ± 0.1 hairpins per gene) and longer hairpins (9.4 ± 0.3 versus 8.4 ± 0.4 basepairs) as compared to representative control reporters present before and after amplification (Figure 5b and 5c). The reporters that were compared (with an absent or a present call) were taken from the same region of expression (same signal intensities).

**Figure 7:**
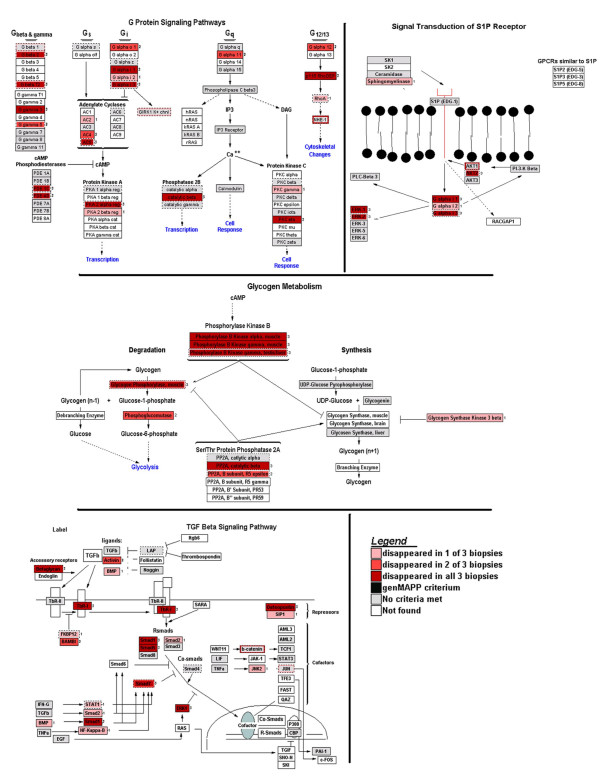
Visualization of low abundance reporters that disappeared after amplification in 1 to 3 LV biopsies (indicated with different shades of red), as compared to the expression profiles found in the parental LVs with the computer application GenMAPP. MAPPs with the highest z-scores in GenMAPP are shown; they represent four major signalling pathways.

Finally, we were able to relate these lost, mainly low-intensity, reporters of all three biopsies to 4 specific major important signalling pathways (Figure 7, Table 2). These pathways had z-scores greater than 1 in MAPPFinder, and included the TGF-beta signalling pathway, the G-protein signalling pathway, the signal transduction pathway of the SIP receptor, and the glycogen metabolism signalling pathway. In those pathways at least 29 % of genes changed. In contrast, false positive reporters never represented more than 5% of the genes in biological pathways available for GenMAPP.

## Discussion

In order to perform a gene expression study on biopsy-material, RNA amplification is necessary. Most studies on RNA amplification and gene expression have until now focused on the optimization of the amplification protocol with diluted high-quality RNA [2]. Stenman et al. [10] proved dilution to be prone to sampling errors because of stochastic distribution of low-abundance transcripts. In addition, applications like biopsies and laser capture material mostly yield RNA that is not of high quality [6,11]. Therefore, we optimized our protocol in the eventual experimental setting, i.e. with undiluted biopsy RNA.

The quality of first-round amplified cRNA is critical for the subsequent reactions (second-round labelling and array hybridization) to succeed. Since these reactions are expensive, it is worthy to be able to check cRNA quality before proceeding. Real-time PCR can serve as a quality control step of first-round amplification, thereby saving time and money by preventing labelling and test chip hybridization of unsuccessfully amplified RNA samples.

To verify the representativeness of the samples used in the current study comparisons were made with data from an unpublished study and a study published in GEO. From this it can be concluded that the quality of the data obtained from our biopsy studies is not largely different from other data on unamplified heart RNA.

Although the small biopsies yielded minimally degraded RNA, we found that the gene expression profiles after amplification were reliable and representative of the gene expression profiles identified in the parental left ventricles of the rat heart. Amplification is known to cause loss of low-expressed reporters [2]. Our results show that this loss is not a random process. We found that false negatives significantly correlated with two kinds of nucleotide strand characteristics. Firstly, in contrast to a study by Gomes et al [4], we found a highly significant correlation with GC content of gene transcripts. This can be explained by the higher affinity bonds between nucleotides G and C as compared to A and T, resulting in difficulties for the DNA polymerase to come in-between 'high GC'-double stranded cDNA in order to make new copies [12]. Secondly, we found that hairpin formation (secondary folding structures that often result from the presence of nucleotide repeats) was correlated significantly to gene loss after mRNA amplification. The sudden hair-pin-kinks in their templates can cause DNA polymerase enzymes to fall off or stop during strand synthesis. Both characteristics will result in sub-optimal amplification of the target strand.

In addition, we proved in another way that loss of gene detection was not a random process. The analyzed biopsies showed a consistent loss of specific low-abundance genes belonging to major important signalling pathways, including the TGF-beta signalling pathway, the G-protein signalling pathway, the signal transduction pathway of the SIP receptor, and the glycogen metabolism signalling pathway.

In summary, reporters lost after amplification of small biopsy material have specific sequence characteristics *and *belong to specific signalling pathways. Although the observed differences in detection might be the result of differences in starting material, it is of importance to know the above described consequences when one has no alternative to using biopsy material. However, differences in detection might also be caused by the different labelling procedures (reverse transcription with dT-T7 for parental LVs versus RT with random primers for the biopsies).

The inability to detect low-expressed transcripts in limited amount of RNA can possibly be overcome by hybridizing all of the labelled cRNA, as suggested by Li et al [2]. When starting with less than 50 ng of total RNA, hybridizing the entire labelled product will increase the amount of detectable transcripts, especially of those that are less abundant.

Since two biopsies cannot be taken from the same place in the LV, we expected a potential large variability between biopsy gene expression profiles, because of regional differences in gene expression or because biopsies could represent different cell types. However, the gene expression results of LV biopsy #4 and 6, taken from parental LV #2, were 96.3 % identical. Even comparison to LV biopsy #2 taken from another parental LV (LV #1), correlated well (92.8 %) although a higher variation in gene expression could be expected. Finally although a considerable extensive amount (21 %) of expression data was lost by the biopsy amplification protocol, the LV biopsies yielded a gene expression pattern that was representative of that of their parental LV for the remaining reporters.

## Conclusion

This study demonstrates that it is possible to amplify RNA obtained from minute biopsies of the rat LV tissue in a reproducible way. Although LV biopsies have been described before in larger animals like dog [13] and sheep [14], our group has first reported on the use of cardiac biopsies taken from rodents [15]. This new biopsy technique shows that it is possible to obtain very small material from rat hearts in-vivo. This study establishes that the gene expression profile obtained from minute LV biopsies is representative for the whole parental LV. However, compared to whole parental LV material, a significant set of gene transcripts were undetectable in the minute LV biopsies. It is notable that the gene transcripts that were lost due to the amplification process were not randomly distributed, but rather had specific sequence characteristics *and *were mainly found in the lower intensity range and represented specific signalling pathways. Importantly, these were signalling pathways involved in hypertrophy and heart failure, which must be taken into account in future biopsy-gene array studies on such animals.

## Methods

### Microarrays

Affymetrix GeneChip^® ^Rat Expression Array 230A (REA 230A), with primarily probe sets against well annotated full-length genes was used for the analysis of gene expression profiles. The array includes a representation of the RefSeq database sequences. Oligonucleotide probes complementary to each corresponding sequence are synthesized *in situ *on the arrays. Eleven pairs of oligonucleotide probes are used to measure the level of transcription of each sequence represented on the GeneChip Rat Expression Set 230.

The GeneChip^® ^Test3 is used to determine the quality of a labelled target prior to its analysis with the GeneChip expression arrays (REA 230A). This test array contains probe sets representing a subset of characterized genes from various organisms and a subset of human and mouse housekeeping genes.

### Animals and samples

Adult 16 weeks old male Wistar rats (body weight 350–400 g) were purchased from Charles River (Maastricht, The Netherlands). Experiments were performed according to the guidelines of the University of Maastricht and were approved by the institutional animal ethics committee, in agreement with the *Guide for the Care and Use of Laboratory Animals *published by the US National Institutes of Health (NIH Publication No. 85-23, revised 1996). The animals were kept on a 12:12-h light-dark cycle in a temperature-controlled (21 ± 2°C) room. During the experiment animals had *ad libitum *access to standard food pellets (Ssniff, Soest, Germany) and water.

Three biopsies of left ventricular (LV) tissue were taken from 2 rats in order to measure the gene expression profile of the biopsies and of the complete left ventricular tissue.

Data from the study by Schweitzer et al. [8] were downloaded from the Gene Expression Omnibus (GEO) [7]; GEO accession no. GSE2690.

### Myocardial biopsies

Fourteen rats were weighed and anesthetized with ketamine (Nimatek^®^, Eurovet, Bladel, The Netherlands, 45 mg/kg i.m.) and xylazine (Xylalin^®^, Ceva Sante Animale, Maassluis, The Netherlands, 5 mg/kg s.c.). The thorax was shaved and the rats were fixed on a surgical table. Body temperature was monitored with a rectal probe and maintained at 37°C using a warming pad and heating lamp. A plastic tube (PE 205) was placed in the trachea and connected to a volume cycled rodent respirator (model 683, Harvard Apparatus, South Natick, MA). Positive pressure respiration was applied at a frequency of 80 strokes/min and stroke volume of 3.5 ml. The skin was incised between the 4^th ^and 5^th ^rib on the left side of the thorax. The underlying pectoral muscles were gently retracted to get access to the intercostal muscles. Using a small pair of tweezers and scissors the intercostal muscles were carefully cut (without damaging the lungs), about 2 mm lateral from the sternum. The heart was exposed by retracting the ribs and the pericardium was opened. Biopsies of left ventricular (LV) tissue were taken from eight rats by pressing a custom-made 0.35 mm (diameter) needle gently into the anterior wall of the heart. The needle was connected to a slowly (150 rpm) rotating handdrill made by the Instrument Services department of our university and advanced 1.3 mm into the left ventricular wall. Care was taken to avoid pressing the needle into the lumen of the ventricle, because then the biopsy may be lost into the blood stream. Three biopsies were taken from each LV. The samples were immediately snap-frozen in liquid nitrogen. Of two biopsied rats, the hearts were excised immediately after biopsy-taking and LVs were snap-frozen in liquid nitrogen, in order to compare gene expression profiles of biopsies to parental LVs. Six animals were sham-operated, i.e. the same surgical procedures were performed but without taking of the actual biopsies. In all rats the chest was closed using 3-0 silk and negative pressure was restored by gently compressing the chest. The pectoral muscles were placed over the wound and the skin was closed with 3-0 silk. After recovery of anaesthesia buprenorphine (Temgesic^®^, Schering-Plough, UK; 0.2 mg/kg s.c.) was given as analgesic. The injection of buprenorphine was repeated the next day in the morning and evening hours.

### Evaluation of left ventricular contractility

LV contractility of 12 rats (6 biopsied and 6 sham operated) was evaluated 14 days after the cardiac biopsy or sham surgery. For this purpose rats were anesthetized with urethane (1.5 mg/g body weight i.p., Sigma, St Louis, USA). Body temperature and respiration were controlled as described above. A 2.0 F high-fidelity catheter tip micro-manometer (SPR671, Millar Instruments, Houston, TX) was inserted through the right carotid artery into the left ventricular cavity. Ventricular pressure was measured and sampled at a rate of 2 kHz. Maximal positive (+ dP/dt) and negative (-dP/dt) pressure development were determined on a beat-to-beat basis and one-second averages were stored on disk. Measurements were performed in baseline conditions and during maximal adrenergic stimulation of the heart. This was achieved by an i.v. ramp-infusion of dobutamine (1.5 to 15 μg/kg.min, Sigma) using a microinjection pump (Model 200 Series, KdScientific, Boston, MA). In this protocol the infusion rate of the dobutamine solution (50 μg/ml) was increased every 2 minutes by 20 μl/min up to 100 μl/min and maximum values in the last 30 seconds of each period were determined.

### RNA isolation

LV biopsies (biopsy#1–6) and their respective LVs (parental LV #1 and #2) were snap frozen in liquid nitrogen before RNA isolation. The two parental LVs were homogenized with a rotor-stator and total RNA was isolated using the RNeasy Mini kit (Qiagen, Valencia, CA), following manufacturer's instructions. Total RNA was isolated from 6 biopsies with the PicoPure^® ^RNA Isolation Kit (Arcturus, Mountain View, CA), according to manufacturer's instructions. Briefly, the frozen biopsy was added to the RNA extraction buffer using a pipette-tip and immediately lysed by pipetting up and down. Then, the lysate was heated at 42°C for 30 minutes, precipitated with 70% ethanol, transferred to a pre-conditioned silica membrane column, and DNA and proteins were removed by a series of wash and elution steps. Total RNA was eluted in 11 μl elution buffer.

The quality of the RNA samples was measured in a 2100 Bioanalyser (Agilent Technologies, Amstelveen, The Netherlands) using the Eukaryote Total RNA Nano and Pico assay, respectively. Total RNA quantity was determined by the NanoDrop^® ^ND-1000 UV-Vis Spectrophotometer (Nanodrop technologies, Rockland, USA).

### RNA amplification

Total RNA isolated from the LV biopsies was amplified for a second round using a protocol largely based on the linear T7-based procedure described by Baugh [16], with some minor modifications, and thereby resembles the current Affymetrix protocol for first round RNA amplification (GeneChip^® ^Two-Cycle cDNA Synthesis, round 1). We used 30 ng of total biopsy RNA in a reverse transcription reaction with 50 pmol oligo(dT)-T7 promotor primer (5'-GGCCAGTGAATTGTAATACGACTCACTATAGGGAGGCGG-(dT)_24 _3'; Affymetrix, Inc., Santa Clara, CA) for first strand cDNA synthesis. For second-strand synthesis, first-strand mRNA fractions cleaved by RNase H served as primers, after which the double-stranded cDNA was purified by phenol:chloroform:isoamyl extraction. In vitro transcription was then mediated by T7 polymerase activity (Ambion, Austin, TX) on the incorporated T7 promotor. The synthesized antisense RNA (aRNA) was purified using RNeasy mini columns (Qiagen, Valencia, CA) and then quantified using the NanoDrop^® ^ND-1000 UV-Vis Spectrophotometer (Nanodrop technologies, Rockland, USA).

### Reverse transcription and real-time quantitative analysis of total RNA and one round- aRNA

Sixty ng of biopsy total RNA and first round-aRNA were reverse transcribed to single-stranded cDNA using 0.5 μg random primers and 100 U Superscript II reverse transcriptase (Invitrogen, Life Technologies, Breda, The Netherlands). Real-time quantitative PCR was performed using Taqman primers and probes designed with Primer Express Software (PE Applied Biosystems, Foster City, CA) (Table 3). We designed exon spanning probes to prevent amplification of possibly contaminating rat genomic DNA. A high-, medium-, and low-abundance transcript was selected in order to address quality and quantity of the RNA.

Optimal PCR conditions were found to be 12.5 μl 2 × PCR Master Mix for Taqman™ assays, with a final concentration of 5 mM MgCl_2_, 300 nM of each primer, 200 nM probe, and 10 ng cDNA-template in a total volume of 25 μl. Amplification and detection were carried out using the ABI Prism 7700 Sequence Detection System (PE Applied Biosystems).

### RNA labelling and hybridization

First round biopsy-aRNA (200 ng) and heart-total RNA (5.8 μg) were biotin-labeled in a separate amplification round. For first strand cDNA synthesis, random primers were used in case of aRNA and oligo(dT)-T7 promotor primers were used in case of total RNA. Second strand cDNA synthesis and biotin-labelling by in vitro transcription were performed following GeneChip^® ^Eukaryotic target labelling assay as instructed by Affymetrix. Length of the labelled cRNA products was assessed by Eukaryote mRNA Nano assay in a 2100 Bioanalyser (Agilent Technologies, Palo Alto, CA, USA). Then 12 μg of the labelled copy RNA (cRNA) products were fragmented following Affymetrix instructions. The fragmented cRNA products were firstly hybridized to GeneChip Test3 gene arrays to assess cRNA quality, and then to Rat Expression Set 230 A (REA230A) GeneChip arrays (Affymetrix), for 16 hours at 45°C following standard Affymetrix protocol.

### Image and data analysis

The gene arrays were scanned by using a Hewlett Packard Genearray Scanner (Hewlett -Packard, Palo Alto, California, USA) using identical parameters for all slides.

From data image files, gene transcript levels were determined with the use of algorithms in the Microarray Analysis Suite Software version 5.0 (MAS 5.0) (Affymetrix). Global Scaling was performed by setting the average signal intensity of each array to a Target Signal of 500. Since the data were scaled, normalization was not necessary and the normalization value was set to 1.0. From each gene array a chip file was created that contained the output from the analysis (signal, detection call and detection p-value).

Expression profiles from two GeneChip gene arrays were compared as follows: one array was designated as the baseline array and another as the experimental array. A comparison chip file was created with the output of the comparison (signal log ratio, change call and change p-value). To determine the most significant changed transcripts a "robust change" analysis was performed which included the metrics detection (present), change (increase or decrease) and signal ratio (1.0 or -1.0).

The chip files were transformed into Excel files and loaded into Spotfire Decision Site (Somerville, MA, USA) to visualize the results of the MAS 5.0 software.

### Publication of microarray data

The gene array data are publicly available in the ArrayExpress database at the European Bioinformatics Institute (EBI) (Accession number: E-AFMX-10).

### Sequence feature analysis

We studied selected structural features of the consensus sequences of the probe sets that were present on the gene array of the parental LVs but absent on the gene array of the amplified LV biopsy-samples. The GC content of all reporters present in both heart-samples was calculated in an automated way as a percentage of the total nucleotides present in the RefSeq sequence (as was used for probe set design) [17] using a custom-made script [18]. The GC content of reporters not detected after amplification was compared to the GC content of reporters present before (whole heart) and after (biopsy) amplification. Hairpin formation of selected disappeared reporters (from the higher intensity range (>3000) and representative controls taken from the same signal intensity region was assessed manually by the program DNAMAN version 4.0 (Lynnon Corporation, Quebec, Canada) and number and length of hairpins were calculated per consensus sequence on the GeneChip.

### Identification of biological processes in array data

To identify biological processes affected by the RNA amplification, the visualisation tool GenMAPP (Gene Map Annotator and Pathway Profiler) version 2.0 [19] was used. This is a generally accessible program for viewing and analyzing gene array data on microarray pathway profiles (MAPPs) representing biological pathways or any other functional grouping of genes [20]. We used all rat MAPPs generated from the Gene Ontology database [21,22], and in addition we used local rat MAPPS generated from the G-protein Coupled Receptor Database [23,24], the KEGG database [25-27] and MAPPs specifically designed for GenMAPP. We imported the gene expression data into the program and dynamically linked them to the MAPPs with a tool called MAPPFinder, that calculates the percentage of genes that meet a user-defined criterion and a z-score [28]. The z-score is a standardized difference-score using expected value and standard deviation of the number of genes meeting the criterion on a MAPP, taking into account MAPP sizes, and it is a measure for differential gene expression.

### Statistical analyses

All data are presented as mean ± standard deviation. Significance was accepted at P <0.05. Analyses were performed using the statistical package SPSS 10.0 (Chicago, IL, USA), employing student's t-test and Mann-Whitney test.

In addition Average Relative Standard Deviations (ARSD) on arrays and Euclidean distances between arrays were calculated using dedicated tools written in C++. Tools are available on the BiGCaT Bioinformatics Tools database [18].

## Authors' contributions

RvH performed the image analysis, the sequence feature analysis and the identification of biological processes in array data. Together with BS she was responsible for the actual writing of the paper. BS performed all the wet-lab activities (mainly concerning the micro-arrays; RNA isolation, RNA amplification, Reverse transcription and real-time quantitative analysis of total RNA and one round- aRNA, RNA labelling and hybridization). She also performed the statistical analysis. Together with RvH she was responsible for the actual writing of the paper. BJ was responsible for the development of the method to get myocardial biopsies from rat in-vivo and initiated the present study. AvE performed the statistical calculations of the comparisons between the different studies. JD was responsible for the animal work. He also took the actual biopsies from the living rats. HS was involved in the design and execution of the microarray experiments described. JS was responsible for the initiation and development of the research program that evaluates changes in gene expression profiles in cardiovascular disease. AvdW optimized the protocol for RNA amplification and gave technical support during amplification experiments. YP played a major role in the design of the wet-lab part of this study. CE was involved in the image and data analysis of the micro array data and gave critical comments on the way things were analyzed. Finally he had an active role in the writing of the paper by critically reviewing and rewriting parts of the manuscript. All authors read and approved the final manuscript.

## Figures and Tables

**Table 1:** Experiment, arrays, ARSD values and average Euclidean distance for the 4256 probe sets present on all arrays and for the 67 Affymetrix normalization probe sets present on all 31 arrays.

		**ARSD**		**Average Euclidean distance**
	
**Study**	**Arrays**	**4256 sets**	**67 norm**	
Amplification (this study)	biopsy (n = 3)	0.023	0.018	1.56
	heart (n = 2)	0.008	0.006	0.66
Amplification 2 (unpublished)	biopsy group1 (n = 4)	0.016	0.013	0.94
	biopsy group2 (n = 6)	0.021	0.016	1.22
	biopsy group3 (n = 4)	0.021	0.017	1.26
Exercise (Schweitzer et al.)	sedentary (n = 4)	0.012	0.010	0.69
	moderate ex (n = 4)	0.011	0.008	0.63
	runners (n = 4)	0.012	0.011	0.70

	weighted average:	0.015	0.012	
	weighted sd:	0.005	0.004	

**Table 2:** List of genes present in parental left ventricles (LVs) but absent in LV biopsies after microarray analysis. The Pathways (MAPPs) with the highest z-scores in MAPPFinder are given in column one with the related genes in those pathways which disappeared after amplification. Column two gives the Affymetrix Probe IDs of the corresponding genes. In column three the number of LV biopsies in which the gene is absent is given. (Three biopsies were used in the experiment so the maximum number is 3).

Pathway/ Gene	Probe ID	# Biopsies (max = 3)
Glycogen Metabolism (12 genes on array; 8 genes changed; 67 %)		
Phosphorylase B kinase alpha, muscle	1369357_at	3

Phosphorylase B kinase gamma, muscle	1387445_at	3
Phosphorylase B kinase gamma, testis/liver	1367788_at	3
PP2A, catalytic beta	1387261_at	3
PP2A, B subunit, R5 epsilon	1387485_a_at	2
Glycogen phosphorylase, muscle	1370982_at	1
Phosphoglucomutase	1369473_at	2
Glycogen Synthase Kinase 3 beta	1370267_at	1

Signal transduction of S1P receptor (17 genes on array; 5 genes changed; 29 %)		

AKT2	1368832_at	3
G alpha i 1	1387505_at	3
G alpha i 2	1367844_at	1
ERK-1	1387771_a_at	3
ERK-2	1369078_at	3

G protein signaling pathways (50 genes on array; 17 genes changed; 34 %)		

G beta 2	1398863_at	3
G beta T2	1398863_at	3
G gamma 3	1373202_at	3
G gamma 5	1398818_at	2
AC2	1367978_at	1
AC4	1368370_at	2
AC5	1368298_at	3
G alpha o 1	1368879_a_at	2
G alpha i 1	1387505_at	3
G alpha i 2	1367844_at	1
PKA 2 alpha reg	1371059_at	3
G alpha 11	1387822_at	2
Phosphatase 2B Catalytic beta	1387261_at	3
PKC gamma	1369089_at	1
PKC eta	1369830_at	3
G alpha 12	1369278_at	2
P115 RhoGEF	1370002_at	3

TGF Beta Signaling Pathway (40 genes on array; 13 genes changed; 30 %)		

Betaglycan	1369219_at	3
TbR-I	1369504_at	3
Activin	1369012_at	2
BMP	1387232_at	1
Smad1	1369174_at	3
Smad5	1369276_at	3
Smad7	1368896_at	2
Smad2	1368214_at	1
ERK1	1387771_a_at	3
NF-Kappa-B	1370968_at	1
JNK2	1368646_at	1
Osteopontin	1367581_a_at	3
SIP1	1368229_at	1

**Table 3:** Sequences of real-time quantitative RT-PCR primers and probes of high-, medium- and low-abundance gene transcripts in rats

Gene	Primer	Sequence (5'→3')	Position
Cyclophilin A (M19533) (high)	F	GGGAGAAAGGATTTGGCTATAAGG	167–190
	Probe	TGAAGTCACCACCCTGGCACATGAAT	219–244
	R	GCCACCAGTGCCATTATGG	249–267

Ribosomal Protein S9 (NM_031108) (medium)	F	CTCGACCAGGAGCTAAAGTTGATT	99–122
	Probe	ACACCTCACGTTTGTTCCGGAGTCCATACT	127–156
	R	TCTTCGCCAGGGTAAATTTGA	163–183

Collagen VI alpha 3 (XM_346073) (low)	F	CAGGAGGACCGAGAGCTCAT	7630–7649
	Probe	ATGCCTTGCAGATCAATAACACAGCAGTAGG	7652–7682
	R	CCCTCCTTGCAGGCAGAAC	7693–7711

The probes were labelled at the 5' and 3' positions with 6-carboxyfluorescein reporter and 6-carboxytetramethylrhodamine quencher, respectively. The position of the primers and probes were annotated according to the sequences derived from GenBank (accession numbers given in parenthesis). F, forward; R, reverse.
